# A healthier approach to clinical trials evaluating resveratrol for primary prevention of age-related diseases in healthy populations

**DOI:** 10.18632/aging.100579

**Published:** 2013-07-20

**Authors:** James M. Smoliga, E. Sage Colombo, Matthew J. Campen

**Affiliations:** ^1^ Institute for Human Health and Sports Science Research, Department of Physical Therapy, High Point University, High Point, NC, USA, 27262; ^2^ Department of Pharmaceutical Sciences, College of Pharmacy, University of New Mexico, Albuquerque, NM, USA, 87131

**Keywords:** biomarker, biosensor, cardiovascular disease, sirtuin, nutraceutical, vascular cell adhesion molecule

## Abstract

In recent years, the wealth of basic science research supporting resveratrol's potential to treat, delay, and even prevent age-related chronic diseases has led to a number of human clinical trials. While such translational research has yielded promising results in clinical populations, recently published conflicting results from studies evaluating resveratrol's potential for primary prevention of chronic disease in healthy / asymptomatic individuals have generated considerable controversy and do not initially appear consistent with findings from animal models. We argue that trials targeting healthy humans are often fundamentally flawed owing to inappropriate use of paradigms only applicable to populations with overt clinical disease and the consequent misleading (typically negative) results can severely retard advancement of drug development. To appropriately perform translational research centered on resveratrol as a primary prevention agent in non-clinical populations, it is critical to utilize study designs which can provide adequate information on clinically relevant outcome measures, avoid paradigms and assumptions from interventions which are specific to clinical populations, and maintain realistic expectations compared to interventions which provide the theoretical maximal response (e.g., caloric restriction and aerobic exercise training).

Treatment with resveratrol, a polyphenol found in red wine, has been suggested as an intervention to prevent chronic disease and promote longevity. Recently, the results from multiple human clinical trials exploring the health impact of resveratrol treatment have emerged, with a general focus on treating individuals with obesity, diabetes, and cardiovascular disease. Basic research in a variety of animal models has shown benefits of resveratrol including increased longevity, amelioration of cardiovascular disease, improved sensitivity to insulin, reduced aging-related neurocognitive decline and neuropathies, and reduced cataract formation through mechanisms largely centered on pathways ultimately related to Sirtuin gene activation [1-3]. In many, but not all cases, the results of these trials are consistent with laboratory animal data, suggesting potential clinical value for resveratrol in these populations [4]. There is also considerable interest in using resveratrol as an agent for primary prevention. Reports of improved healthspan in laboratory models [5] have spurred the commercialization of resveratrol supplements [6], which are marketed for improving health and preventing chronic disease in healthy populations, rather than treating existing disease. Several recent papers have reported the effects of resveratrol treatment in clinically healthy populations; however, conflicting results have fueled appropriate skepticism towards resveratrol's clinical potential.

Yoshino [7] concluded that resveratrol treatment “does not have beneficial metabolic effects in non-obese, postmenopausal women with normal glucose tolerance.” Likewise, Poulsen [8] stated that lack of significant findings in otherwise healthy obese individuals “raises doubt about the justification of resveratrol as a nutritional supplement in metabolic disorders” and that “it is likely that a certain degree of baseline metabolic abnormalities is a prerequisite to benefit from resveratrol treatment.” Agarwal [9] concluded “resveratrol may have protective effects against atherosclerosis in individuals who would not be considered high risk with the current screening criteria” and Timmers [10] noted that resveratrol “induces metabolic changes in obese humans, mimicking the [beneficial] effects of calorie restriction.” Naturally, conclusions from each study were appropriate to reported results, which were driven by differences in study design, cohorts, and clinical outcomes. Discrepancies in results between studies highlight the unique challenge of evaluating resveratrol efficacy in a healthy population - how does one define clinical improvement in individuals who are already clinically healthy at baseline?

## Appropriate Clinical Outcome Markers Are Necessary for Appropriate Conclusions

Identifying outcome measures for interventional trials in populations with overt clinical disease is relatively straightforward, as patients' signs, symptoms, and abnormal biomarker values can be monitored at specific intervals and improvement can be clearly identified (e.g., resolution of signs, values moving towards the normal healthy reference ranges). In animal models, resveratrol treatment can be started early in life and effectiveness of primary prevention interventions can be directly measured through serial biomarker measurements, age at onset of disease, maximal lifespan, and post-mortem pathological evaluations (e.g., [2]). Comprehensive nutritional primary prevention studies in humans are more difficult to perform due to time constraints, costs, and limitless confounding factors [11] which can lead to a bias towards negative findings [12]. Thus, various biomarkers associated with risk for chronic disease are often utilized as a surrogate to predict the development of pathology or risk for disease-associated mortality (e.g. [13]) and anti-aging therapeutics are evaluated in part through their ability to treat age-related pathologies [14].

**Figure 1:**
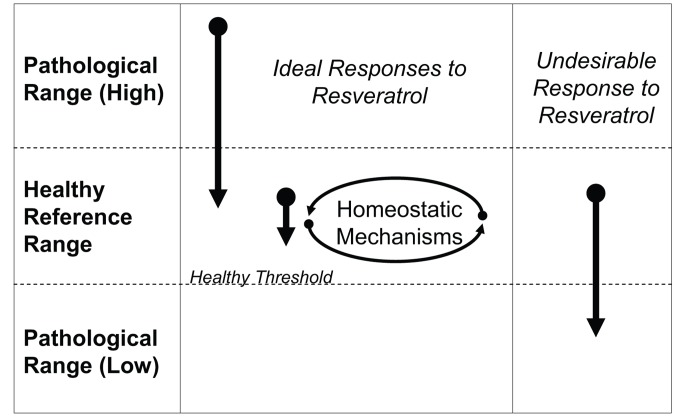

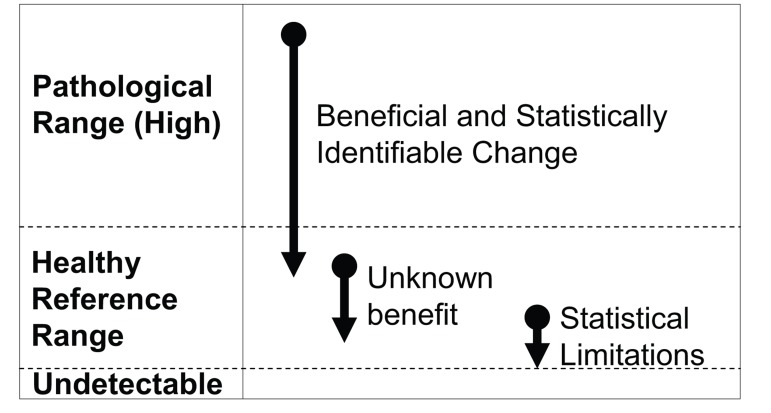
Unique challenges in selecting outcome measures which are responsive to resveratrol treatment in healthy individuals (**A**) When there is a continuum for an outcome measure such that values above or below a reference range are pathological (e.g., fasting glucose or blood pressure), it is advantageous for resveratrol treatment to produce an effect in the pathological population. However, in healthy individuals, homeostatic control mechanisms should prevent these values from dropping below a certain healthy threshold to create pathology. As such, these types of outcome measures are likely to exhibit a limited range of response to resveratrol. (**B**) When plasma biomarkers are above normal reference range, there is often known benefit in reducing them. However, in the healthy population, most biomarkers associated with pathology (e.g., inflammatory cytokines) are found at very low concentrations. The physiological benefits of further reducing these biomarkers remains unknown, and the limited possible range for response creates statistical limitations.

Research paradigms which are typically applied to populations with overt clinical syndromes or disease are not necessarily relevant to asymptomatic, healthy populations. Perhaps the clearest example of this is based on the tenet that if pathology causes a change in an outcome measure in one direction (e.g., increased blood glucose or blood pressure), a change in the opposite direction is indicative of improved health (e.g., decreased blood glucose or blood pressure). Indeed, the paradigm that resveratrol is efficacious in clinical populations, including obese individuals, individuals with cardiometabolic dysfunction, and elderly individuals is well supported through clinical trials [15-18]. However, when resveratrol clinical trials have applied this paradigm to healthy populations, the results are often disappointing (e.g. [7, 8]). In the absence of pathological deviations from healthy reference range for a given outcome at baseline, therapeutic-induced changes in said outcomes are difficult to interpret. Reductions in many parameters, such as electrolytes, glucose, or blood pressure, may be deleterious if they fall below “normal” values (Figure 1a). On the other hand, a reduction in plasma C-reactive protein, as an example of an inflammatory marker, does not have a clear interpretation if the basal levels were already in a low-risk category (Figure 1b). Thus, the recently reported very small, yet statistically significant decreases in TNFα and IL-6 in healthy athletes following resveratrol treatment is of unknown clinical value[19]. Only longitudinal studies of substantial duration that incur natural progression of disease (or biomarkers thereof) would be of value to highlight a retarded progression of age-related diseases induced by therapeutics (e.g., similar to those using statin drugs [20, 21], fish oil [22], or vitamin supplements [23] for prevention of cardiovascular disease). However, cross-sectional studies suggest that the natural history for many biomarkers of age-related disease could require decades of follow-up for clinical trial studies, which is typically not feasible.

**Figure 2:**
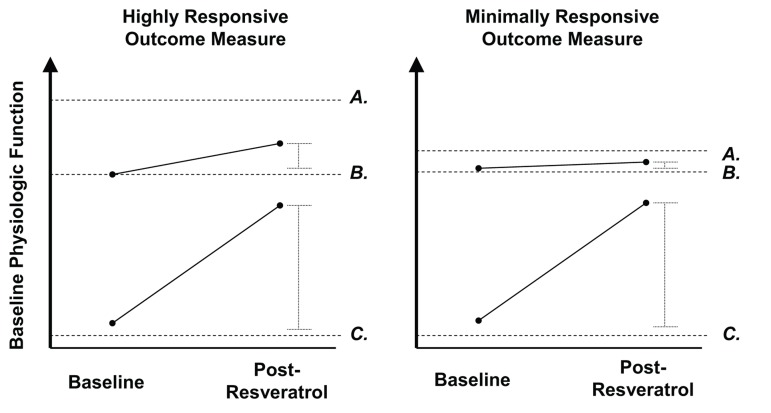
Model comparing expected changes in highly responsive vs. poorly responsive outcome measures to resveratrol treatment in pathological and healthy individuals The y-axis represents baseline physiologic function, with each dashed line corresponding to the following categories: Letters represent the following: (**A**) Maximal physiological response attainable through optimal stimulus (i.e., high intensity exercise training, caloric restriction). (**B**) Healthy normal. (**C**) Metabolic dysfunction (e.g., diabetes mellitus). For an outcome measure which resveratrol treatment produces a large effect in a pathologic individual, it is expected to show a smaller effect in an already healthy individual. In healthy individuals, highly responsive outcome measures (left) will show a large response to an optimal physiologic stimulus (e.g., high intensity aerobic exercise, caloric restriction), but may have a smaller, yet detectable response, to resveratrol treatment. However, outcome measures which are minimally responsive to such optimal stimuli in the healthy population would be expected to have an undetectable response to resveratrol treatment.

Given that resveratrol mimics effects of caloric restriction [24-28] and exercise training [29, 30] across model species, it is appropriate to use these two interventions as benchmarks to define the theoretical maximal response to resveratrol treatment. It may be unreasonable to expect even the largest tolerable dose of any nutraceutical alone to provide an equivalent metabolic response to a 40% caloric restricted diet [31] or replicate the multiple cardiovascular and metabolic adaptations resulting from aerobic exercise [32] (e.g., 30% increase in SIRT1 activation following 6 weeks of high-intensity interval training [33]). Thus, it would be expected that resveratrol treatment would elicit at least a somewhat lower magnitude of response compared to those achieved through “best case scenarios” of the two reference interventions, with the greatest responses likely observed in the elderly or individuals with cardiometabolic dysfunction (Figure 2). If resveratrol treatment can mimic the effects of exercise training, albeit at a reduced magnitude, athletes should serve as a model for many of the expected physiological responses.

Indeed, athletes do exhibit extreme values in certain measurements (e.g., high maximal aerobic capacity, low body fat) and exercise training does substantially reduce the risk of cardiovascular and metabolic disease [32] and thus may be regarded as an anti-aging intervention [34]. Yet, many relevant measures of physiologic function in athletes remain unchanged compared to the general healthy population. For instance, resting arterial blood pressure and HOMA is not significantly reduced in most competitive athletes [35] and hypertension is not uncommon in athletic populations [36]. It is not surprising that these particular measurements do not severely decline in response to resveratrol treatment in normotensive normoglycemic individuals (i.e., individuals who do not have “loss of homeostasis” [37]), given that hypotension and hypoglycemia are indicative of disrupted homeostasis and would be pathologic, rather than advantageous. Thus, if the “best case scenario” (years of exercise training) does not significantly impact these variables, it seems unlikely that a short period of resveratrol treatment would influence these in healthy individuals whose baseline values are also already within a normal reference range. Thus, when resveratrol does not influence tightly regulated biomarkers in the asymptomatic healthy population, it should not be interpreted as a negative finding (e.g., [7, 8]). In fact, deviation of parameters such as glucose or blood pressure from “normal values” would easily be construed as adverse events.

Likewise, some outcome measures are not fully representative of overall function. This is exemplified in evaluating general cardiovascular fitness or endurance performance through measuring maximal aerobic capacity (VO_2max_). In mice, resveratrol promotes mitochondrial biogenesis and improves endurance performance [38] and thus, VO_2max_ has served as an outcome measure in one resveratrol human trial [10] and will likely appear in future trials as a measure of overall cardiometabolic function. However, VO_2max_ reflects the interdependent functioning of multiple systems and, given the serial nature of oxygen transport, a major improvement at one site without parallel improvements elsewhere will have limited influence on VO_2max_ [39]. While resveratrol treatment does induce some physiologic adaptations, such as increased blood flow [40-42]) and enhanced skeletal muscle mitochondrial function [10] in humans, other variables are still likely to limit aerobic function (e.g., maximal cardiac output [43]). Endurance performance is also influenced by other variables independent of VO_2max_, such as movement economy and fractional utilization [44], and factors which are only likely to adapt to exercise training itself (e.g., neuromuscular activation patterns [45]). Because exercise is the only intervention that can directly increase absolute VO_2max_ in healthy individuals, it should not be considered a negative finding when resveratrol fails to enhance a multifaceted measurement that all other dietary (e.g., [46]) and very few pharmaceutical (namely erythropoietin [47, 48]) interventions also fail to improve.

## Dosage, Inter-Individual Variability, and Interactions

As research in non-clinical populations becomes more common, disparity in dosing protocols and clinical endpoints will likely continue to cause conflicting findings. The wide range of daily resveratrol dosage used in clinical trials for healthy individuals (75mg [7] to 5000mg [49]) would be expected to result in different clinical responses. Brown [49] confirmed this, demonstrating 2500mg to be more effective than both lower (500mg and 1000mg) and higher dosages (5000mg) in reducing plasma IGF-1 concentrations. Though 1000mg resveratrol did not alter IGF-1 concentrations, it was sufficient to reduce IGFBP-3 concentrations. This demonstrates that there may not be a single optimal dose of resveratrol, but rather the ideal dose may vary depending on the target outcome measures, which is not uncommon for various drugs (e.g., low vs. high dosages of corticosteroids to induce anti-inflammatory vs. immunosuppressive responses [50]). Further, that only relatively high dosages are able to influence at least some variables counters Yoshino's [7] argument that 75mg of resveratrol should be sufficient to observe a physiological response in healthy individuals. *In vitro* studies have demonstrated time of exposure influences the cellular response to resveratrol (e.g., [51]), and thus it is possible that short-duration peaks can produce very different effects than sustained elevations in plasma resveratrol concentration. Thus, it is quite difficult to compare the physiologic response of a resveratrol treatment that maintains high plasma concentrations (i.e., Poulsen's 500mg three times per day [8]) to protocols in which there is greater circadian fluctuation in plasma resveratrol concentration (i.e., any of the once daily dosing protocols).

It is accepted that genetic factors can account for inter-individual responses to drug therapy, including both efficacy and toxicity, and the issue of individualized medicine based on pharmacogenomics remains an active topic of discussion in the medical community [52, 53]. Additionally, inter-individual variability in gene expression and single nucleotide polymorphisms, such as those related to sirtuins, AMPK, and NAD(P)H oxidase, in the general population may be related to metabolism [54-57], and cardiovascular health [58-61], and response to cardiometabolic drug targets [62], and could therefore influence response to resveratrol. Likewise, physiologic responses to resveratrol and other natural products may also be expected to depend on a complex array of multiple individual factors which ultimately influence baseline status for sirtuin activity, NAD+ levels, and other molecular pathways associated with resveratrol's mechanisms of action (e.g., age [63-65], sex [63], race [66], diet [67, 68], exercise practices [64]). There is also emerging evidence that inter-individual variability in the human gut microbiota can have a major influence on resveratrol metabolism, which likely has consequences for both bioavailability and physiologic responses [69]. As such, it is critical that studies involving healthy humans control either control for as many of these confounders as possible to determine the effects of resveratrol on a specific population of interest, or ensure that there is adequate sample size to overcome inter-individual variability and detect overall responses when examining the general population and to allow subgroup analyses whenever possible.

There is evidence that resveratrol's bioavailability can be enhanced when it is administered with other polyphenols [70], which has led to some studies incorporating resveratrol into a polyphenol matrix (e.g. [9, 71]). While polypharmaceutical treatments do not allow one to differentiate between the effects of resveratrol versus the complete matrix, it can be argued that maximizing clinical utility is more important than differentiating the physiological effects of one small molecule from another. Limitless possibilities in formulation of resveratrol supplements create challenges in generalizing conclusions regarding the clinical efficacy of resveratrol [72]. Therefore, some attempt should be made to collect data using a standardized dose and / or a standardized set of outcome measures whenever possible. When possible, clinical trials should utilize sufficient sample size to study multiple dosages of resveratrol (e.g., 250mg vs. 500mg per day), different administration schedules to achieve a total dose (e.g., 500mg once per day vs. 250mg twice per day), different formulations (e.g., resveratrol alone vs. resveratrol within a specified matrix), or some combination thereof. Admittedly, this will not provide answers to all of the unknowns related to optimal dosage, but may shed light on what dosage protocol appears most advantageous, and whether different outcome measures require different dosage protocols.

To better understand the relationships between dosage, bioavailability, and physiological response, research studies should consider plasma bioavailability in their analysis. In other words, grouping individuals with high and low plasma bioavailability together does not allow for a true assessment of physiologic response to resveratrol. Indeed, it is possible that individuals who appear to be “non-responders” to resveratrol may not be absorbing as much bioactive compound as those who have more pronounced responses or may demonstrate a different metabolite profile (e.g., [73-75]). In the absence of such pharmacokinetic data, it is inappropriate to conclude that resveratrol was not effective when it may have produced clinically meaningful effects in some individuals. When outcome measures which can realistically respond to treatment (as described earlier) remain unchanged, the particular resveratrol treatment protocol used (e.g., orally administered tablet containing a specified amount of resveratrol at given time intervals) may be deemed ineffective in the population studied, however, it is premature to conclude that resveratrol itself does not produce physiological effects in humans. It is hoped that novel delivery methods or resveratrol congeners which overcome limitations in absorption and bioavailability may reduce the wide inter-individual variability in bioavailability and considerably increase the magnitude of physiologic response [76, 77].

The general paradigm for resveratrol human clinical trials has been that of using resveratrol as a stand-alone treatment, rather than a molecule which may potentially enhance the effects of other interventions. While there is merit in finding low-cost, safe alternatives to pharmaceutical products for treating existing disease, it may be unrealistic to find a nutraceutical that is a complete alternative to healthy behaviors (i.e., proper dietary and exercise practices). For instance, the French Paradox strongly suggests that red wine consumption lowers one's risk for cardiovascular disease [78], but one would be hard pressed to find a clinician who recommends moderate wine consumption as an equal alternative to aerobic exercise. Likewise, it follows that the benefits of resveratrol should not be viewed in isolation, but rather as one of many components in primary prevention. Thus, future clinical trials should explore whether resveratrol can interact with other supplements, pharmaceuticals, or even diet and exercise interventions to accelerate or amplify health benefits. Indeed, such beneficial interactions have been demonstrated in animal models [79-81].

## Novel Biosensors for Studying Responses to Anti-Aging Interventions in Healthy Individuals

Clinical trials have generally examined changes in multiple biomarkers and evaluated them independently of one another. This assumes that changes in each biomarker occur separately, and thus the physiological effects are generally considered in isolation. Further, the biomarkers measured between studies are inconsistent, and therefore it is possible that biomarkers which change in response to resveratrol treatment are not even measured in some studies. The effect size may be small for many of the biomarkers measured, especially in healthy individuals whose values are already within normal reference ranges, as consistently observed across multiple plant-derived food and beverage interventions [82]. However, it is possible that small changes consistent across multiple biomarkers may have major clinical implications. Thus, it may be especially useful to interpret biomarkers holistically.

**Figure 3:**
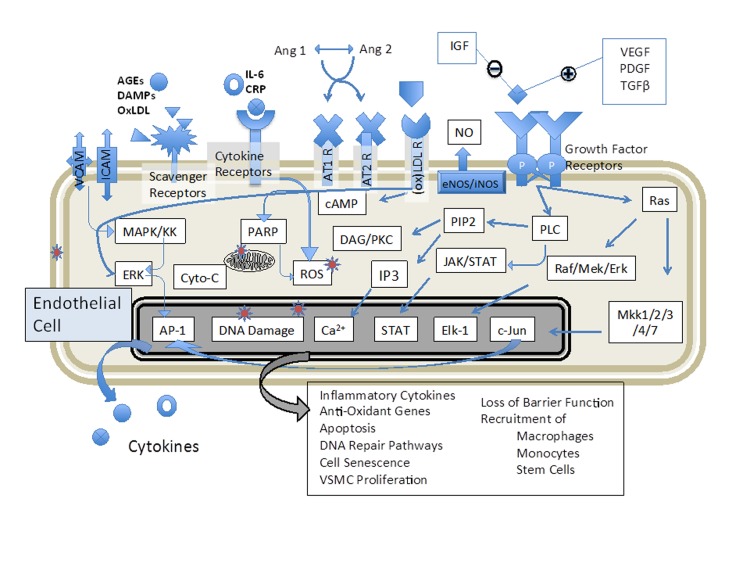
Biosensing of plasma components allows an unbiased approach to determine if therapeutics have altered the endothelial environment in an anti-inflammatory manner Endothelial cells respond positively or negatively to a wide range of serum components, including cytokines, hormones, growth factors, prostaglandins and other metabolites; however, conducting even a massive bioinformatic screening for all potential factors does not link the various measured components to a cumulative bioactivity. As the development of atherosclerotic disease is a decades-long process, maintaining the blood in a low-inflammatory phenotype is beneficial for the long term. Healthy individuals may not show overt signs of vascular disease progress in the relatively short period of a clinical trial for a nutritional supplement or diet, but their plasma composition can change rapidly and the relative inflammatory potential (and therapeutic efficacy) can be assessed with this or related techniques.

We feel it is not possible to model a theoretically near-infinite array of biomarkers and N-factorial interactions and further interpret how complex interactions between each coalesce to cause *in vivo* physiologic changes. Chronic vascular disease is tightly associated with the condition of the endothelium [83-85]. As endothelial cells detect injury, they recruit inflammatory cells to aid in the resolution of insults. However, chronic mild inflammatory processes result in substantial remodeling and occlusive, fragile and coagulative phenotypes in susceptible regions, such as the aortic arch, carotids, coronary and cerebrovascular arteries [83, 85]. We postulate that small modifications of plasma composition, caused by diet, infectious disease or environmental insults, can lead to endothelial cells responses that, over many decades, contribute to such pathological vascular remodeling associated with aging. In the short term, however, such insults are modifiable and to a limited extent reversible. Nutraceuticals that can offset the negative impacts of environmental toxins may have a dramatic life-long benefit in this paradigm. Thus, in the setting of chronic cardiovascular disease, there is good reason to combine *in vitro* techniques with human specimens, allowing primary human cells to provide net inflammatory readouts of the cumulative impacts of serum components (Figure 3).

**Figure 4:**
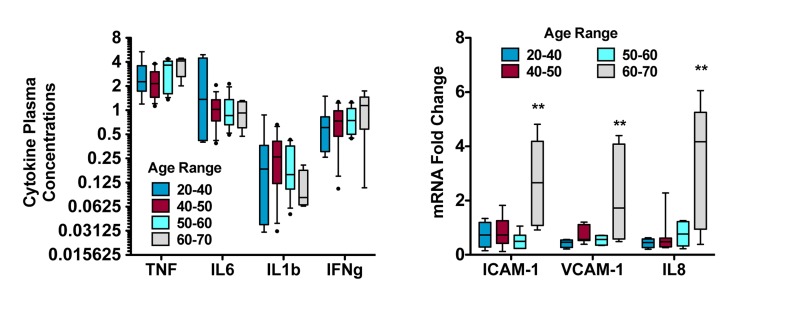
Assessment of aging-related plasma inflammatory potential reveals greater complex than presence of cytokines Replotted baseline data (before treatment) from subjects studied in a month-long trial of resveratrol treatment [9]. Plasma cytokines, including tumor necrosis factor-alpha (TNF), interleukin-6 (IL6), interleukin-1beta (IL1b) and interferon gamma (IFNg), were not significantly altered as a function of age (left). However, serum from those subjects applied to primary human coronary artery endothelial cells elicited responses that increased dramatically in individuals 60-70 years of age. These findings, along with the results of the resveratrol study and environmental health research, strongly suggest that the complex milieu of the circulation - and its ultimate inflammatory potential - should be addressed in functional, holistic assessments.

This is the approach taken by Agarwal [9], in which baseline and post-treatment plasma from human subjects was incubated with human primary coronary endothelial cells. Gene expression of canonical response elements associated with atherosclerosis (ICAM and VCAM) and inflammation (IL-8) decreased in cells incubated with post-treatment plasma from healthy human subjects treated with resveratrol, but not those treated with placebo. Authors concluded that the inflammatory potential of the circulation was reduced by resveratrol. It is noteworthy that the magnitude to which expression of endothelial cell adhesion molecules and chemokines increased from air pollutant exposure [86] is comparable to the reduction afforded by resveratrol treatment [9]. Moreover, this paradigm is translational and can be conducted in animal studies to better ascertain mechanistic pathways or validate the relationship between acute response and chronic disease [87]. Interestingly, when examining baseline inflammatory potential in the serum from subjects on this study [9], a very apparent and significant trend for increasing plasma inflammatory potential was noted in subjects over 60 years old (Figure 4). These subjects were otherwise healthy and undiagnosed in terms of cardiovascular, renal or liver disease, and their plasma cytokine profiles were unremarkable compared to younger subjects (Figure 4). While follow -up was not possible in these subjects, these findings suggest an important age-related increase in plasma inflammatory potential that may reflect ongoing or early-stage disease (such as sub-clinical chronic kidney disease) and may also explain associations between organ-specific diseases (ie, lung [88, 89] or kidney disease [90]) and atherosclerosis.

In Agarwal et al [6], the mechanism of action for the reduced plasma inflammatory potential is unclear. Certainly, it stems from either a reduction in generation of pro-inflammatory factors or an enhancement in the clearance of such factors. Basic studies of resveratrol note broad spectrum benefits in terms of increased insulin sensitivity, mitochondrial numbers, increased capacity for vasorelaxation, and reduction in a number of plasma factors [1, 2, 38]. Interestingly, while the plasma factors reduced by resveratrol treatment, including triglycerides and fasting glucose, may be considered drivers of vascular disease, they may also reflect overall liver function, which in turn may be processing many other components out of the plasma [91]. Certainly in the setting of metabolic syndrome, hepatic function lowers circulating glucose and results in reduced levels of advanced glycation end-products that are deleterious for vascular function [91, 92]. Liver function may reflect an important acute readout of nutraceutical benefit in otherwise healthy individuals, as this organ is responsive to acute environmental changes (e.g., [93, 94]), ranging from dietary/pharmaceutical input to pathological states.

This method of treating primary cells with serum/plasma from research subjects provides an advantage over *in vitro* work involving treatment of cells with drugs or nutraceuticals, in that cultured cells are exposed to conditions representative of those occurring *in vivo* (i.e., all components of the plasma, multi-organ involvement in plasma processing, physiologically attainable concentrations of resveratrol, physiologically accurate resveratrol metabolite profile), which results in a more complete and interpretable functional response. Additionally, direct *in vitro* treatment with resveratrol, and likely numerous other nutraceutical compounds, often requires exceedingly high concentrations to elicit responses that are coherent with *in vivo* outcomes [95]. These observations highlight the value of biosensors in clinical research and emphasize the need for novel paradigms which bridge the gap from “bench to bedside” in evaluating resveratrol's utility.

While our research has focused on endothelial cells and vascular disease, other groups have focused on the impact of the systemic milieu that is the serum on other cell types with an emphasis on aging, growth, and senescence. Efforts from the de Cabo lab have explored directly the impacts of serum from calorie restricted animals and humans on cultured hepatocytes, observing reduced rate of growth and increased survival in the face of oxidative stress [96, 97]. In a recent related study, the effects of various forms of exercise training were tested in endothelial cell lines, revealing variable impact on cellular response to oxidative stress, growth and aging [98]. The collected findings reported to date have been in healthy human subjects and reflect minor perturbations that, in theory, contribute to the chronic advancement of inflammatory vascular disease. Though we infer that such changes are modest and likely reversible, such approaches may be of greater value than biomarkers induced only in the setting of major clinical disease (e.g., CRP) or under tight homeostatic control (e.g., blood pressure).

## Conclusions

As long as some human clinical trials continue to produce positive results, resveratrol will remain a popular candidate for the prevention and treatment of chronic diseases. If one searches for a natural compound which consistently transforms ordinary individuals into “superhumans” with optimal physiologic function, inducing similar physiologic adaptations to those observed from caloric restriction or exercise training, resveratrol and other known substances are bound to disappoint. However, if clinical trials are designed such that sufficient sample sizes and study durations are combined with realistic clinical outcome variables, appropriate conclusions may be reached. Only long-term epidemiological studies and meta-analyses can provide more definitive answers on resveratrol's effectiveness as a primary prevention measure to reduce the incidence, delay the onset, or decrease the severity of chronic diseases. Until then, researchers should be prudent in selecting outcome measures that are sensitive enough to respond to short-to-moderate term resveratrol treatment in healthy individuals. When possible, clinical trials should use multiple formulations or dosages to better determine the optimal administration protocols to achieve the most powerful clinical effects. In addition to exploring the effects of stand-alone resveratrol interventions, it is also important to investigate whether resveratrol can further enhance clinically validated treatments, including existing pharmaceutical treatments and exercise training. Most importantly, assessing factors that contribute to chronic diseases in a sensitive and holistic manner may greatly improve our understanding of the value of resveratrol for primary prevention of cardiometabolic diseases.

Conflicting findings between basic science and human clinical trials, and between different clinical trials, for resveratrol treatment are due to major differences in research protocols, including inappropriate outcome variables and ineffective dosing protocols, which preclude valid assessment of physiological response within humans Biomarkers which are highly responsive to treatment in individuals with chronic disease (eg, blood pressure, insulin sensitivity) are not likely to be as sensitive to interventions in healthy humans, due to normal homeostatic control mechanisms, and therefore should not be used as clinical endpoints in primary prevention studies in non-clinical populations.

The theoretical maximal response for clinical endpoints to optimal treatments (e.g., exercise training, caloric restriction) must be determined to appropriately interpret response to resveratrol treatment, as these responses will vary depending on the health and age of the cohorts studied.

As optimal dosing of resveratrol may vary for different outcome measures, it is not appropriate to make generalized conclusions regarding resveratrol's clinical utility in healthy individuals until considerable uncertainties regarding optimal dosing protocols and factors influencing bioavailability are addressed, and its interactions with other primary prevention interventions are thoroughly explored.

Novel methods that incorporate *in vitro* techniques into human clinical trials, such as whole cell biosensors, can provide a more holistic evaluation of physiologic response.
